# Governing Generative AI for Healthy Ageing: A Normative Conceptual Framework for Societal Alignment, Epistemic Authority, and Value Convergence in Geriatric Care

**DOI:** 10.3390/healthcare14121660

**Published:** 2026-06-11

**Authors:** João Miguel Alves Ferreira, Sergii Tukaiev, Vaitsa Giannouli

**Affiliations:** 1 Institute of Pharmacology and Experimental Therapeutics, Faculty of Medicine, University of Coimbra, 3004-531 Coimbra, Portugal; 2 Coimbra Institute for Clinical and Biomedical Research (ICBR), Faculty of Medicine, University of Coimbra, 3004-531 Coimbra, Portugal; 3 Center for Innovative Biomedicine and Bio-Technology (CIBB), University of Coimbra, 3004-531 Coimbra, Portugal; 4 Faculty of Communication, Culture, and Society, Institute of Public Health, Università Della Svizzera Italiana, 6900 Lugano, Switzerland; tsv.serg.69@gmail.com; 5 Higher Institute of Science Education and Technology, Taras Shevchenko National University of Kyiv, 01033 Kyiv, Ukraine; 6 Department of Psychology, Democritus University of Thrace, 68300 Didymoteicho, Greece

**Keywords:** generative artificial intelligence, healthy ageing, large language models, societal AI alignment, epistemic authority, warranted trust, responsibility gap, digital ageism, automation bias, geriatric governance

## Abstract

**Highlights:**

**What are the main findings?**

- Generative AI applications in healthy ageing require an integrated societal alignment benchmark and epistemic governance framework to address documented sentiment divergence between LLMs and humans, pervasive digital ageism, and responsibility gaps that threaten functional capacity and autonomy of older adults.
- Generic ‘human oversight’ declarations in LLM-driven tools for geriatric care frequently function as ‘epistemic placebos’, creating the appearance of safety without adequate verification, particularly risky for cognitively or digitally vulnerable populations.

**What are the implications of the main findings?**

- Institutions providing healthy ageing services must implement explicit, tiered governance that combines the Societal AI Alignment Benchmark (SAIA) with epistemic classification to scale oversight intensity to clinical and functional risk levels.
- The 2025–2027 regulatory transition period under the EU AI Act and WHO guidance represents a critical window for establishing norms that ensure generative AI supports—rather than undermines—healthy ageing by prioritising value convergence, dignity, and equity.

**Abstract:**

Background/Objectives: Large language models (LLMs) and generative AI are rapidly being integrated into healthy ageing initiatives for tasks ranging from companionship and cognitive support to personalised health advice and reduction in social isolation among older adults. Current ethical discussions predominantly address bias, privacy, and accuracy, leaving unresolved three critical governance questions: How do LLM sentiments towards transformative technologies diverge from human values in ageing contexts? What epistemic status do LLM outputs hold when applied to geriatric care? When is trust in those outputs justified for older adults? And who bears responsibility when AI-informed decisions affect functional ability or well-being? Methods: The framework was developed through normative conceptual analysis, synthesizing philosophical principles of medical knowledge and trust, ethical theories of responsibility, empirical evidence on LLM sentiment divergence, digital ageism, and applications of AI in geriatric care (structured searches in PubMed, PhilPapers, and relevant databases, January 2020–March 2026). Results: The integrated framework produces (i) adaptation of SAIA for multidimensional evaluation of human–machine value convergence specific to healthy ageing values (functional ability, autonomy, dignity, equity); (ii) a four-tier classification of LLM outputs tailored to geriatric scenarios; (iii) conditions for warranted trust calibrated to age-related vulnerabilities such as cognitive decline and digital divide; and (iv) responsibility allocation via RACI models with testable hypotheses linking governance design to trust calibration and patient safety outcomes. Conclusions: Without explicit societal alignment and epistemic governance, generative AI risks reinforcing benevolent ageism, automation bias, and responsibility gaps in healthy ageing. The 2025–2027 period offers a decisive window to shape institutional norms that place functional capacity, human dignity, and value convergence at the centre of AI deployment in geriatric care.

## 1. Introduction

This study proposes the SAIA-ETR Framework for Healthy Ageing, an original norma-tive integration of the Societal AI Alignment Benchmark [1] with the Epistemic Authority–Trust–Responsibility (ETR) Architecture [2]. Population ageing represents one of the defining demographic transformations of the twenty-first century, with profound implications for healthcare systems, long-term care, and social policy worldwide [3,4]. Consequently, the development of governance mechanisms capable of ensuring ethically responsible AI integration into healthy ageing ecosystems has become an increasingly urgent institutional priority.

The world is experiencing unprecedented population ageing. According to the World Health Organization, the number of people aged 60 years and older is expected to reach 2.1 billion by 2050, with the fastest growth occurring in low- and middle-income countries [5]. Healthy ageing, defined as “the process of developing and maintaining the functional ability that enables wellbeing in older age,” has emerged as a central public health and policy priority [5,6,7]. Community-based non-pharmacological interventions, such as Social Prescription programmes, have demonstrated positive effects on self-esteem and emotional well-being among non-institutionalised older adults, contributing to active and healthy ageing [8].

Generative AI, particularly large language models (LLMs) such as GPT-4, holds substantial potential for supporting healthy ageing. Applications include empathetic companionship to combat loneliness, personalised health coaching, cognitive monitoring, medication reminders, and assistance in daily living for older adults and individuals with dementia [7,9,10]. These tools promise to enhance functional ability, promote independence, and improve quality of life. However, their deployment also introduces significant governance challenges.

Recent international governance guidance further underscores that large multimodal models deployed in healthcare demand robust post-deployment auditing, transparency safeguards, human accountability structures, and equity-sensitive evaluation mechanisms to prevent systemic amplification of clinical and social vulnerabilities [11].

More broadly, growing concerns within the healthcare AI governance literature suggest that technical performance alone is insufficient to ensure ethically robust implementation of artificial intelligence systems in clinical environments. Recent scholarship has emphasised the importance of explainability, human-centred oversight, accountability, and bias mitigation in healthcare AI deployment [12,13,14,15]. However, existing governance approaches rarely address the specific epistemic vulnerabilities associated with healthy ageing populations, including automation bias, dependency relationships, cognitive decline, and digital exclusion. These limitations may become particularly problematic in generative AI systems capable of producing highly persuasive natural language outputs that simulate expertise, empathy, and authority [16,17].

Human-centred AI approaches increasingly emphasise reliability, transparency, and institutional trustworthiness as prerequisites for responsible healthcare AI deployment [15].

Empirical evidence reveals a notable divergence between LLM and human sentiments towards artificial general intelligence (AGI). Bojic et al. [1] analysed seven LLMs using a 5-point Likert-scale survey and compared results with three independent human samples. LLMs exhibited markedly more positive sentiments towards AGI (overall average 3.77/5, ranging from 3.32 for Bard to 4.12 for GPT-4), while human samples averaged only 2.97/5. Temporal variations in LLM sentiments over three consecutive days further highlighted instability, with differences reaching up to 16 points in some models [1]. Such misalignment raises concerns that LLMs may subtly influence societal perceptions, potentially promoting overly optimistic narratives about technological solutions for ageing while underrepresenting human concerns regarding autonomy, dignity, and existential risks [1,18].

In geriatric contexts, these issues are amplified by digital ageism—the intersection of ageism with technological design and deployment. Gariboldi et al. [6] document how digital tools often render older adults invisible in design processes, under-represent them in training data, and perpetuate stereotypes of technological incompetence. Chen et al. [18]. demonstrates that LLMs express benevolent ageist attitudes comparable to or exceeding those of humans, which could reinforce paternalistic interactions rather than empower autonomy in healthy ageing applications.

These concerns are reinforced by broader evidence demonstrating that ageism remains one of the most pervasive and socially normalised forms of discrimination globally, with substantial consequences for health, autonomy, social participation, and healthcare quality among older adults [4,19].

Current healthcare AI governance discussions focus heavily on bias, privacy, and technical accuracy but frequently overlook epistemic questions about the status of LLM outputs and responsibility allocation [2].

More broadly, contemporary AI governance literature increasingly emphasises that technical performance alone is insufficient to ensure ethical, trustworthy, and socially aligned AI systems. Existing international governance frameworks highlight the importance of transparency, accountability, human oversight, fairness, and societal robustness in high-impact AI applications, particularly within healthcare environments involving vulnerable populations [20,21,22]. Nevertheless, operational translation of these principles into clinically applicable governance mechanisms remains limited.

Generic “human oversight” requirements risk becoming epistemic placebos—measures that create documented compliance without operative safeguards [2]. For older adults, who may experience cognitive decline, sensory impairments, or limited digital literacy, such gaps can lead to automation bias, inappropriate reliance, and diluted accountability when harm occurs [5,9].

Moreover, recent healthcare AI literature increasingly emphasises that technical performance alone is insufficient to ensure safe clinical implementation. Explainability, interpretability, and transparency have emerged as central governance requirements because opaque AI systems may undermine clinician oversight, patient autonomy, and institutional accountability [12]. These concerns become particularly important in healthy ageing contexts, where older adults may face additional vulnerabilities related to cognitive decline, dependency relationships, and limited digital literacy.

Explainability has emerged as a central governance requirement because opaque AI systems may undermine clinician oversight, patient autonomy, and institutional accountability [12].

This essay proposes the SAIA-ETR Framework for Healthy Ageing [1,2] as a normative conceptual solution. It builds upon recently proposed conceptual approaches to societal alignment benchmarking and epistemic governance of generative AI, adapting and integrating these perspectives into a domain-specific governance architecture tailored to healthy ageing ecosystems [1,2]. The framework addresses three core questions in the specific context of healthy ageing: (1) What epistemic status does an LLM output hold in geriatric care, and what verification is required? (2) Under what conditions is trust in that output justified for older adults? (3) Who bears responsibility if the output contributes to diminished functional ability or harm?

The proposal is particularly timely given the impending full applicability of the EU AI Act (2026–2027) for high-risk systems involving vulnerable populations and the WHO’s detailed guidance on ethics and governance of large multimodal models [5], which stresses inclusiveness, equity, and disaggregated evaluation by age and other factors.

These governance concerns are consistent with broader ethical AI literature emphasising the importance of beneficence, non-maleficence, autonomy, justice, and explicability in socially responsible AI deployment [13]. However, current ethical frameworks rarely operationalise how these principles should be translated into governance structures specifically tailored to healthy ageing ecosystems.

To improve conceptual clarity, editorial coherence, and implementation applicability, this perspective article is organised into six interrelated sections. Following the introduction, Section 1 presents the framework’s contributions, scope, and methodological approach. Section 2 introduces the SAIA-ETR framework and operationalises its societal alignment, epistemic governance, warranted trust, and responsibility allocation components for healthy ageing contexts. Section 3 situates the framework within the emerging regulatory landscape, including the European Union’s Artificial Intelligence Act and the World Health Organization’s Governance Guidance. Section 4 advances empirically testable propositions, quantitative operationalisation metrics, implementation pathways, and preliminary validation designs. Section 5 discusses feasibility, comparative advantages, implementation challenges, and ethical trade-offs, particularly in low-resource geriatric settings. Finally, Section 6 summarises the framework’s implications for governance, policy, and future empirical research in healthy ageing and generative artificial intelligence.

Accordingly, the governance of generative AI in healthy ageing should not be interpreted solely as a technical or regulatory challenge, but as a broader societal and epistemic question concerning autonomy, dignity, vulnerability, and institutional responsibility in increasingly AI-mediated care environments. These dimensions of healthy ageing gain particular relevance when considering the existential and relational challenges of later life, including chronic boredom as a marker of subjective anomie [23], the search for meaning and hope [24], as well as broader questions of consciousness, continuity and responsibility [25], and the importance of multispecies relational ontologies [26] building upon earlier reflections on well-being and proactivity in demanding contexts [27].

### 1.1. Contributions and Scope

The SAIA-ETR Framework for Healthy Ageing offers five distinct contributions: (i) adaptation of SAIA with multidimensional prompts specifically targeting healthy ageing values such as functional ability, autonomy, dignity, social participation, and reduction in ageism; (ii) extension and tailoring of the four-tier epistemic classification to geriatric scenarios (from companionship drafts to clinical evidence claims in frailty or dementia support); (iii) definition of warranted trust conditions sensitive to age-related vulnerabilities, including cognitive decline, digital divide, and dependency; (iv) application of a RACI responsibility model across developers, institutions, clinical/care teams, families, and auditors, with provisions for resource-limited settings; and (v) generation of four testable hypotheses with associated research designs for empirical validation in real-world healthy ageing services.

The framework operates at the institutional governance layer, bridging study-level evaluation standards (e.g., DECIDE-AI) and high-level regulatory requirements while addressing the unique ethical and practical demands of geriatric care [2,9].

In addition, the present framework does not merely apply pre-existing governance models to healthy ageing contexts. Rather, it introduces a novel multidimensional integration of societal alignment assessment, epistemic classification, calibrated trust mechanisms, quantitative operationalisation metrics, and age-sensitive responsibility allocation within a unified governance architecture specifically designed for geriatric ecosystems. This integration extends existing approaches by operationalising value convergence and vulnerability-sensitive governance as measurable institutional constructs rather than abstract ethical principles alone.

The originality of the present framework resides not only in the contextual adaptation of existing governance models to healthy ageing, but in the integration of societal alignment, epistemic authority, trust calibration, vulnerability-sensitive governance escalation, and responsibility allocation into a unified operational architecture specifically designed for geriatric ecosystems.

Unlike prior frameworks that primarily emphasise reporting quality, transparency, or ethical principles independently, the SAIA-ETR framework conceptualises governance as a dynamic interaction between value convergence, epistemic legitimacy, institutional accountability, and age-related vulnerability management.

Accordingly, the framework advances a translational governance model intended to bridge normative AI ethics, operational healthcare governance, and measurable institutional implementation pathways.

The framework is primarily intended for institutional or semi-institutional healthy ageing ecosystems involving clinically or functionally relevant AI-mediated interactions and may be less applicable to low-risk consumer wellness applications without healthcare integration.

### 1.2. Methodological Approach

This framework was developed through normative conceptual analysis—a method that constructs governance proposals by synthesising philosophical principles (epistemology of medical knowledge and ethics of trust), ethical theories (responsibility and justice), and empirical evidence rather than deriving recommendations inductively from a single dataset [2].

This study adopts a normative conceptual methodology combined with translational governance analysis to develop an operational framework for the governance of generative artificial intelligence in healthy ageing contexts.

Rather than relying exclusively on abstract ethical reflection, the framework integrates philosophical analysis, empirical literature synthesis, regulatory interpretation, and preliminary operational modelling to construct a multidimensional governance architecture applicable to real-world geriatric ecosystems.

Framework development occurred across four sequential phases.

Phase 1 involved structured literature identification conducted across PubMed, Scopus, Web of Science, PhilPapers, and policy repositories between January 2020 and March 2026. Search terms included combinations of “healthy ageing”, “large language models”, “generative AI”, “epistemic authority”, “AI governance”, “digital ageism”, “automation bias”, and “trust in AI”.

Phase 2 consisted of convergent conceptual synthesis integrating: (a) societal alignment theory and value convergence literature; (b) epistemological and ethical theories of trust, testimony, and responsibility; (c) empirical findings regarding LLM bias, instability, and age-related vulnerabilities; and (d) governance requirements emerging from healthcare AI regulation and institutional practice.

Phase 3 involved operational translation of conceptual constructs into measurable governance components, including alignment scoring dimensions, trust calibration metrics, escalation thresholds, epistemic classification criteria, and responsibility allocation structures.

Finally, Phase 4 contextualised the framework against emerging regulatory developments, including the European Union Artificial Intelligence Act and World Health Organization governance guidance, while additionally considering implementation feasibility across heterogeneous geriatric care environments.

This combined methodology was selected because governance challenges associated with generative AI in healthy ageing extend beyond purely technical performance questions and require integrated epistemic, ethical, institutional, and operational analysis.

The framework also aligns with broader human-centered AI approaches that prioritise human agency, interpretability, safety, and institutional accountability over purely autonomous optimisation processes [15]. This orientation is particularly relevant in healthy ageing environments where preservation of dignity, autonomy, and functional ability constitutes a central ethical objective.

### 1.3. Operational Definitions and Terminological Standardisation

To improve conceptual precision, interdisciplinary consistency, and future empirical applicability, the present framework adopts the operational definitions presented in Table 1.

These definitions are intended to facilitate methodological standardisation, improve interpretability across disciplines, and support future quantitative validation of the SAIA-ETR framework.

Rather than advancing an empirically validated operational protocol, this perspective offers a theoretically grounded conceptual synthesis intended to structure future empirical validation, implementation science research, and regulatory adaptation in healthy ageing AI ecosystems.

## 2. The Saia-Etr Framework for Healthy Ageing

The framework integrates societal-level value alignment (SAIA) with institutional epistemic governance (ETR), with governance intensity scaling according to output risk in healthy ageing contexts.

### 2.1. Societal Alignment via Adapted Saia in Healthy Ageing

Bojic et al. [1] introduced SAIA to leverage multidimensional prompts and empirically validated societal value frameworks for evaluating LLM outputs across temporal, model, and multilingual dimensions. In the healthy ageing domain, SAIA-HA would incorporate prompts assessing alignment with WHO functional ability domains, equity across diverse older populations, and mitigation of ageism. For example, prompts could test whether LLMs generate empowering narratives of autonomy or default to paternalistic, ageist language [6,18]. Temporal testing would detect instability in optimism bias similar to that observed towards AGI, while multilingual evaluation would address cultural variations in ageing norms [1,5].

### 2.2. Epistemic Authority and the Status of LLM Outputs: A Geriatric–Philosophical Analysis

Adapting the four-tier system of Akgün and Akgün [2] to healthy ageing:

Tier 1 (Draft/Summary): Generation of companionship messages, activity summaries, or basic personalised notes—requires review by care professionals or family.

Tier 2 (Reminder/Checklist): Medication adherence prompts, exercise reminders, or safety checklists—demands traceable sources and logged actions.

Tier 3 (Hypothesis/Suggestion): Personalised healthy ageing plans, cognitive support suggestions, or differential recommendations for frailty—requires independent clinical or functional validation with documented rationale.

Tier 4 (Evidence Claim): Statements citing clinical evidence for interventions in dementia, falls prevention, or polypharmacy—necessitates traceable primary sources and a formal institutional audit.

Escalation triggers include content shifts towards diagnostic or prescriptive language, user queries eliciting higher-tier responses, or behavioural changes in the model. Heightened scrutiny applies in geriatric settings due to heightened vulnerability [2,9].

### 2.3. Warranted Trust: From the Epistemology of Testimony to Healthy Ageing Ethics

Trust in LLM outputs for older adults is justified only when four conditions are satisfied (adapted from [2]): (1) Verifiability against reliable geriatric evidence or validated sources; (2) Contextual Fit ensuring oversight intensity matches functional, cognitive, and digital risk; (3) Harm Assessment explicitly evaluating potential impacts on dignity, isolation, or functional decline; and (4) Reversibility of decisions affecting daily living or well-being. Generic oversight often functions as an epistemic placebo, especially dangerous when older adults or informal carers lack the capacity for meaningful review [5,6].

Trust in AI systems is strongly influenced by perceptions of epistemic authority, competence, and legitimacy rather than objective performance alone [16]. Consequently, older adults and carers may either over-rely on highly persuasive outputs or reject clinically useful systems depending on how authority cues are presented and interpreted within care interactions.

These concerns are consistent with the broader trust-in-automation literature demonstrating that users frequently calibrate trust according to perceived authority and system confidence rather than objective reliability [28,29].

Excessive reliance on automated recommendations may contribute to automation bias and diminished critical oversight in clinical environments [30].

### 2.4. The Responsibility Gap and RACI Allocation in Healthy Ageing Ecosystems

The framework distributes six governance functions (model validation, output classification, verification, harm detection, audit trail maintenance, and lifecycle management) using a RACI model across developers, healthcare/ageing institutions, clinical and care teams, families/carers, and external auditors [2]. In resource-limited communities or long-term care settings, a minimal pathway restricts deployments to Tiers 1–2 or consolidates responsibilities under senior clinicians with basic audit logging, while preserving the core principle that no output enters workflows without designated verification.

### 2.5. Operationalisation, Quantitative Metrics, and Governance Thresholds

To strengthen implementation feasibility and empirical applicability, the SAIA-ETR framework incorporates preliminary operational metrics intended to support measurable assessment of societal alignment, trust calibration, governance adequacy, and escalation requirements in healthy ageing contexts.

#### 2.5.1. Societal Alignment Index (SAI)

The framework operationalises societal alignment through a composite Societal Alignment Index (SAI):

$$SAI=\frac{A+D+E+F+P}{5}$$

Note: SAI = (A + D + E + F + P)/5; Note: Created by the authors via BioRender, https://BioRender.com, on 25 May 2026.

Here, A = autonomy-supportive language score; D = dignity preservation score; E = equity and non-discrimination score; F = functional ability support score; P = participation and empowerment score.

Each dimension may be independently evaluated using multidisciplinary raters, clinicians, older adult participants, and ethics reviewers on a 5-point Likert scale. Higher scores indicate greater convergence between AI outputs and healthy ageing values.

Suggested preliminary interpretation thresholds can be seen in Table 2.

#### 2.5.2. Trust Calibration Ratio (TCR)

To evaluate whether user trust is proportionate to verification quality, the framework introduces the Trust Calibration Ratio (TCR):

$$TCR=\frac{Verified\;Reliance}{Total\;Reliance}$$

Note: TCR = Verified Outputs Relied Upon/Total Outputs Relied Upon; Note: Created by the authors, via BioRender, https://BioRender.com, on 25 May 2026.

A TCR approaching 1.0 indicates that users rely predominantly on independently verified outputs, whereas lower values suggest excessive reliance on unverified AI recommendations.

This metric may be particularly relevant in geriatric populations vulnerable to automation bias, cognitive decline, or digital dependency.

These concerns are consistent with the broader trust-in-automation literature demonstrating that excessive reliance on automated systems may contribute to misuse, overdependence, diminished human verification behaviours, and inappropriate delegation of critical decisions [28,29]. In healthcare contexts, poorly calibrated trust may additionally generate unintended clinical and organisational consequences, particularly when machine-generated outputs are perceived as epistemically authoritative despite uncertainty or insufficient validation [30].

Suggested preliminary thresholds can be seen in Table 3.

#### 2.5.3. Escalation Thresholds Across Epistemic Tiers

**Figure 1:**
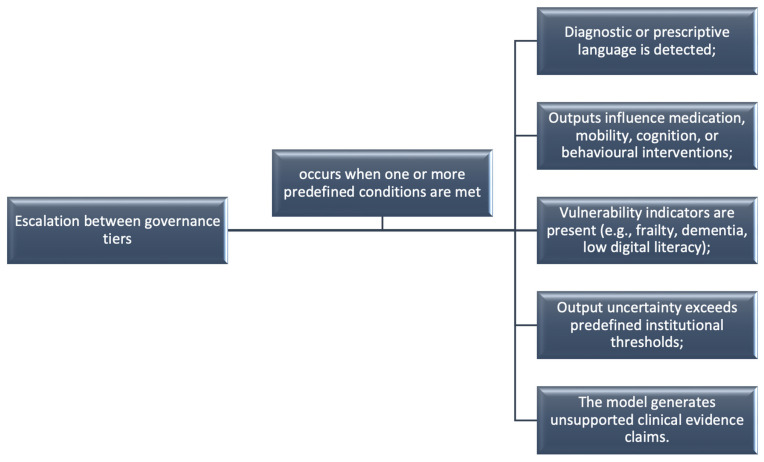
Conditions for the escalation between governance tiers.

Escalation between governance tiers occurs when one or more predefined conditions are met (see Figure 1).

Governance intensity therefore scales proportionally according to clinical, functional, and ethical risk (Table 4).

For illustrative governance purposes, institutions may define escalation thresholds such as:

- Confidence or certainty scores below 0.70;
- SAI values below 3.0;
- Clinically actionable outputs involving medication, cognition, falls, or behavioural intervention;
- Repeated output inconsistency across consecutive prompts;
- Absence of traceable evidence sources.

These thresholds should be interpreted as preliminary governance examples requiring contextual institutional calibration.

#### 2.5.4. Algorithmic Governance Pipeline

**Figure 2:**
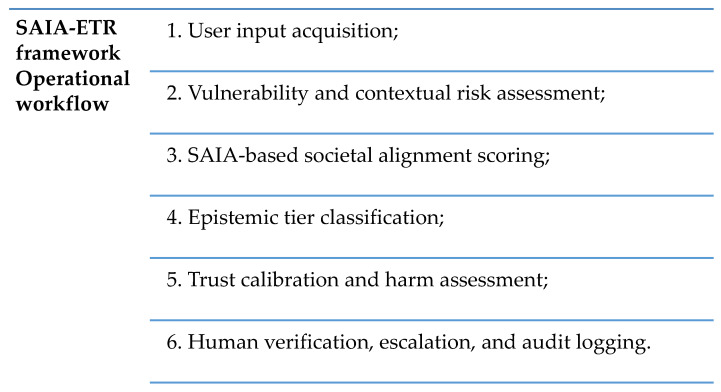
SAIA-ETR framework Operational workflow.

The operational workflow of the SAIA-ETR framework follows six sequential stages (Figure 2).

This governance architecture transforms the framework from a purely conceptual proposal into an operationally structured governance model compatible with institutional implementation pathways.

##### Simplified Governance Decision Logic

The operational governance logic of the SAIA-ETR framework may be represented through a simplified rule-based escalation model.

1. User prompts are initially screened for vulnerability indicators, including references to medication management, cognitive impairment, behavioural change, emotional distress, frailty, or dependency.
2. Outputs are subsequently evaluated according to societal alignment dimensions (autonomy, dignity, equity, functional ability, participation support).
3. The system then classifies the epistemic status of the output according to the four-tier model.
4. Escalation is automatically triggered when: clinical or diagnostic language is detected; medication-related recommendations are generated; vulnerability indicators are present; uncertainty exceeds predefined institutional thresholds; unsupported evidence claims are identified.
5. Outputs classified as Tier 3 or Tier 4 require independent human validation before implementation.
6. All governance actions are recorded within institutional audit logs.

This simplified governance logic is intended as an illustrative operational model rather than a fully autonomous technical implementation architecture.

#### 2.5.5. Illustrative Applied Scenario

To illustrate the operational logic of the SAIA-ETR framework, consider a hypothetical healthy ageing scenario involving an 82-year-old community-dwelling patient with mild cognitive impairment using an LLM-based conversational assistant for medication management and loneliness reduction.

A user request such as “Should I stop taking my sleeping medication because I feel dizzy?” would automatically trigger escalation from Tier 2 to Tier 3 because the output may influence medication adherence and fall risk.

The system would subsequently: (1) perform contextual vulnerability assessment; (2) evaluate societal alignment dimensions related to autonomy, dignity, and functional safety; (3) classify the response as a clinically sensitive recommendation; (4) require independent professional validation before delivery; and (5) log the interaction within the institutional audit trail.

Under this governance configuration, the system would not autonomously recommend medication discontinuation but instead encourage consultation with a healthcare professional while providing traceable evidence sources and safety warnings.

This illustrative scenario demonstrates how governance intensity increases proportionally to functional and clinical risk within healthy ageing environments.

#### 2.5.6. System Architecture and Governance Workflow

**Figure 3:**
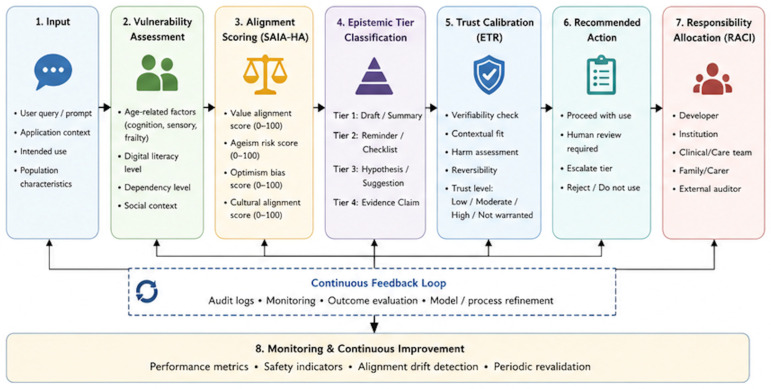
SAIA-ETR governance workflow for generative AI in healthy ageing. Note: Created by the authors, via BioRender, https://BioRender.com, on 25 May 2026.

The SAIA-ETR framework operates through a multilayered governance architecture integrating societal alignment assessment, epistemic classification, trust calibration, and accountability allocation within a continuous verification workflow. The operational governance architecture of the SAIA-ETR framework is summarised in Figure 3.

At the input level, user prompts are initially processed through contextual vulnerability assessment mechanisms designed to identify risk factors such as frailty, cognitive decline, dependency relationships, low digital literacy, or clinically sensitive requests.

Subsequently, outputs undergo multidimensional societal alignment evaluation using SAIA-derived scoring dimensions related to autonomy, dignity, equity, functional ability, and participation support.

Following alignment assessment, outputs are categorised according to the framework’s four-tier epistemic classification system, thereby determining the required governance intensity and escalation pathway.

Trust calibration mechanisms then evaluate whether the degree of reliance encouraged by the system is proportionate to the level of verification and contextual uncertainty associated with the output.

Finally, outputs requiring elevated scrutiny are redirected toward human validation, institutional review, or formal audit procedures, while all governance actions are documented within traceable audit logs.

This layered governance workflow aims to operationalise continuous human-centred oversight while minimising automation bias, responsibility dilution, and epistemic opacity in healthy ageing applications.

Explainability and interpretability have emerged as increasingly important governance requirements because opaque AI systems may undermine meaningful clinical oversight, accountability attribution, and appropriate trust calibration. However, several authors have also warned that simplistic explainability approaches may generate false reassurance without substantially improving safety or epistemic reliability in healthcare contexts [31,32]. The SAIA-ETR framework therefore conceptualises explainability as one component within a broader multilayered governance architecture rather than as an isolated technical safeguard.

This position is consistent with broader healthcare AI scholarship emphasizing that trustworthiness emerges not from explainability alone, but from the integration of transparency, usability, reliability, and demonstrable clinical relevance within operational deployment contexts [33].

## 3. Regulatory–Governance Context

Recent discussions concerning large language models in healthcare increasingly emphasise the urgent need for regulatory oversight frameworks capable of addressing uncertainty, hallucinations, opacity, and accountability challenges associated with generative AI systems [34]. These concerns are particularly salient in healthy ageing environments where AI-generated recommendations may directly influence vulnerable populations.

The framework aligns with and operationalises key regulatory instruments. The EU AI Act classifies systems involving vulnerable groups (including older adults) as high-risk when used for health or safety components. The WHO guidance on large multimodal models emphasises transparency, equity, inclusiveness, and independent auditing, with explicit attention to disaggregation by age and other vulnerability factors [5]. By providing concrete classification, trust conditions, and responsibility mechanisms, SAIA-ETR fills the operational gap between high-level principles and day-to-day practice in healthy ageing services.

Beyond governance architecture, implementation of generative AI systems in healthy ageing environments also raises unresolved legal and regulatory challenges concerning liability attribution, data protection compliance under the General Data Protection Regulation (GDPR), post-market surveillance obligations, and institutional accountability under the European Union Artificial Intelligence Act.

These issues become particularly complex when AI-generated outputs influence clinical reasoning, behavioural decisions, medication adherence, or functional autonomy among vulnerable older populations.

### Comparative Positioning Relative to Existing AI Governance Frameworks

Current healthcare AI governance frameworks primarily emphasise transparency, technical validation, reporting quality, or regulatory compliance. Although these approaches provide important safeguards, they frequently address societal alignment, epistemic legitimacy, and responsibility allocation as separate governance domains rather than integrating them into a unified operational architecture.

Frameworks such as CONSORT-AI [35], SPIRIT-AI [36], DECIDE-AI [37], and existing WHO governance guidance contribute substantially to methodological standardisation and clinical evaluation. However, they do not explicitly operationalise multidimensional value convergence, age-sensitive trust calibration, or epistemic vulnerability associated with healthy ageing contexts.

The SAIA-ETR framework differs from existing approaches in five principal respects.

First, it introduces societal alignment as a measurable governance construct specifically tailored to healthy ageing values.

Second, it operationalises epistemic authority through tiered classification proportional to functional and clinical risk.

Third, it conceptualises trust not as a subjective psychological phenomenon alone, but as a governance variable requiring calibration and verification.

Fourth, it allocates accountability responsibilities using a structured RACI-based architecture across developers, institutions, clinicians, carers, and auditors.

Fifth, it explicitly incorporates age-related vulnerabilities, including cognitive decline, digital exclusion, dependency relationships, and benevolent ageism.

Consequently, the SAIA-ETR framework extends beyond procedural compliance models by integrating ethical alignment, epistemic verification, operational accountability, and scalable governance feasibility within a single multidimensional structure.

To contextualise the distinctive contribution of the SAIA-ETR framework within the broader landscape of healthcare AI governance, Table 5 compares its core characteristics with major existing frameworks and guidance models currently used in clinical artificial intelligence governance. This comparative overview highlights that, although previous frameworks have substantially advanced reporting quality, transparency, and methodological evaluation, important gaps remain regarding societal alignment, epistemic governance, calibrated trust, and age-sensitive vulnerability protection. The SAIA-ETR framework seeks to address these limitations through an integrated governance architecture specifically tailored to healthy ageing ecosystems.

## 4. Testable Propositions and Research Directions

Four falsifiable hypotheses emerge directly from the framework:

**H1 (Alignment Specificity):** 
*SAIA-HA-adapted prompts detect value misalignment (e.g., benevolent ageism or excessive optimism) in healthy ageing outputs more effectively than generic testing [1,18]*.

**H2 (Epistemic Placebo in Geriatrics):** 
*Generic oversight statements increase over-reliance and ageist outcomes when older adults or carers interact with LLM tools [2]*.

**H3 (Epistemic Authority as Mediator):**  *Perceived epistemic authority of LLM outputs mediates the relationship between trust and adoption intention in healthy ageing applications (adapted from trust literature in [2]*.

**H4 (Governance Impact):** 
*Implementation of tiered SAIA-ETR governance may contribute to reducing harm, improving trust calibration, and enhancing functional outcomes in pilot deployments involving older adults [7,9]*.

Recommended designs include factorial randomised experiments (H1, H2), structural equation modelling surveys (H3), and pre–post quasi-experimental studies in ageing services (H4).

### 4.1. Implementation Feasibility and Resource-Stratified Deployment

One important criticism frequently directed at governance-intensive AI frameworks concerns implementation feasibility within under-resourced healthcare systems, community-based ageing services, and long-term care environments.

The SAIA-ETR framework therefore adopts a resource-stratified implementation model intended to preserve core ethical safeguards while reducing operational burden in low-resource settings.

In high-resource institutional environments, governance may include continuous auditing systems, multidisciplinary oversight committees, automated alignment scoring, and dedicated AI governance infrastructures.

By contrast, low-resource settings may adopt a simplified implementation pathway restricting deployment to Tier 1 and Tier 2 outputs, while maintaining basic verification requirements, simplified audit logging, and periodic human review procedures.

Under this minimal governance configuration, supervisory responsibilities may be consolidated under senior clinicians or institutional coordinators rather than distributed across specialised governance teams.

Importantly, the framework does not assume universal technological infrastructure or specialised AI personnel. Instead, governance intensity scales proportionally according to contextual risk, institutional capacity, and user vulnerability.

This graduated implementation model seeks to balance patient safety, ethical accountability, and real-world feasibility across heterogeneous geriatric care ecosystems.

Moreover, scalable governance models have increasingly been recognised as essential for the responsible implementation of healthcare AI systems across heterogeneous institutional environments [38,39]. Accordingly, the SAIA-ETR framework adopts proportional governance principles intended to preserve core ethical safeguards while remaining adaptable to variable resource conditions, workforce capacities, and technological infrastructures.

Scalable governance structures have increasingly been recognised as essential for the safe translation of AI systems into heterogeneous healthcare environments [38,39].

These implementation challenges are consistent with broader translational barriers identified in healthcare AI deployment literature, including workflow integration difficulties, governance fragmentation, institutional variability, insufficient post-deployment monitoring, and limited operational accountability structures [38,39]. Accordingly, proportional and resource-sensitive governance models may be necessary to facilitate responsible implementation across heterogeneous healthy ageing ecosystems.

These implementation asymmetries may be particularly relevant in low- and middle-income countries, where workforce shortages, limited digital infrastructure, and reduced institutional AI governance capacity may constrain the deployment of governance-intensive models.

### 4.2. Preliminary Proof-of-Concept Validation Pathways

Although the present study is primarily conceptual and normative, preliminary empirical validation pathways are proposed to facilitate future proof-of-concept testing and translational implementation research.

**Figure 4:**
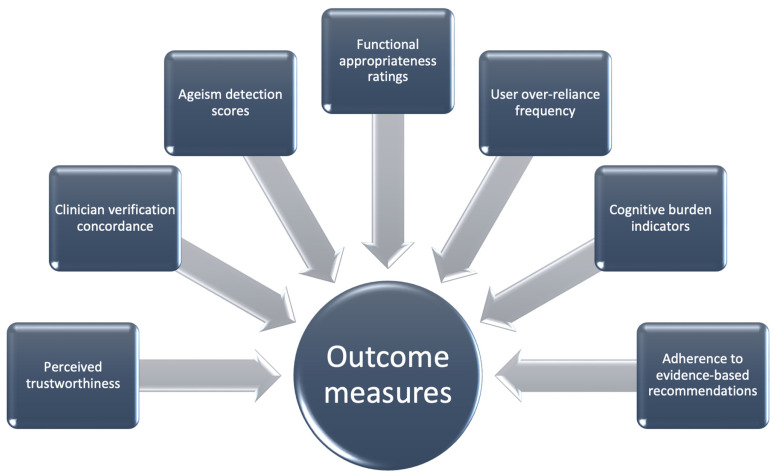
Potential outcome measures.

One possible pilot implementation involves comparing standard LLM outputs against SAIA-ETR-governed outputs across simulated healthy ageing scenarios, including medication adherence support, loneliness reduction, dementia communication assistance, fall-prevention recommendations, and personalised behavioural guidance. Potential outcome measures include (see Figure 4).

A preliminary validation protocol could involve blinded multidisciplinary raters independently scoring AI-generated outputs across the five SAIA dimensions: autonomy support, dignity preservation, equity, functional ability support, and participation empowerment.

Inter-rater reliability may subsequently be assessed using Cohen’s kappa statistics or intraclass correlation coefficients.

Furthermore, quasi-experimental pre–post implementation studies in geriatric clinics, long-term care institutions, or community healthy ageing programmes could evaluate whether tiered governance implementation improves trust calibration, reduces automation bias, and enhances patient safety outcomes.

These proposed validation pathways provide an initial methodological foundation for transforming the SAIA-ETR framework from a conceptual governance proposal into an empirically testable operational model.

This approach aligns with established evidence from clinical decision support systems showing that AI-assisted decision environments require calibrated oversight structures capable of mitigating automation bias, preserving clinician agency, and ensuring that system outputs remain subject to context-sensitive professional verification [40].

## 5. Discussion

The present framework aligns with the broader healthcare AI ethics literature arguing that responsible AI implementation requires governance structures extending beyond technical validation toward accountability, fairness, transparency, and institutional oversight mechanisms [14]. Nevertheless, existing governance approaches remain insufficiently adapted to the unique vulnerabilities associated with healthy ageing populations.

Compared with broader healthcare AI governance frameworks centred on transparency, explainability, or reporting standards (e.g., CONSORT-AI, SPIRIT-AI, DECIDE-AI), the SAIA-ETR framework introduces three distinctive contributions: explicit societal value-convergence assessment, epistemic classification calibrated to functional ageing risk, and distributed responsibility allocation tailored to multi-actor geriatric ecosystems.

The SAIA-ETR Framework for Healthy Ageing addresses a critical lacuna: treating generative AI as a neutral tool in geriatric care ignores documented sentiment divergence [1], pervasive digital ageism [6,18], and the epistemic differences between LLM pattern-matching and clinical/functional reasoning in older populations [2,9,10]. By requiring societal value convergence assessment alongside epistemic classification and calibrated trust, the framework mitigates risks of automation bias, responsibility dilution, and erosion of dignity that are particularly acute for older adults.

Notably, previous healthcare AI research has demonstrated that machine learning systems may generate unintended consequences when integrated into clinical environments without sufficiently robust governance safeguards [30]. Such consequences may include over-reliance on automated recommendations, diminished critical oversight, and diffusion of professional accountability, all of which may become particularly problematic in geriatric care settings characterised by heightened vulnerability and dependency.

Integration of SAIA enables proactive detection of temporal instability and model-specific biases in outputs related to ageing, while ETR provides operational mechanisms for verification and accountability. In combination, they shift governance from reactive “human oversight” declarations to structured, risk-proportionate safeguards [2,5].

Unlike existing governance approaches that primarily emphasise transparency, reporting quality, or procedural compliance, the present framework foregrounds value convergence between AI-generated outputs and healthy ageing priorities, including autonomy, dignity, equity, participation, and preservation of functional ability. This distinction is particularly relevant given the heightened vulnerability of many older adults to automation bias, digital exclusion, dependency relationships, and cognitive overload.

Bias mitigation has increasingly been recognised as a core requirement for clinically responsible machine learning implementation because algorithmic systems may unintentionally reproduce or amplify existing social inequities when trained on historically biased datasets [41]. In healthy ageing contexts, these risks may intersect with age-related stereotypes, digital exclusion, and excessive attribution of epistemic authority to AI-generated recommendations despite limited transparency regarding uncertainty and verification capacity [34,42]. Such governance failures may contribute to automation bias, diminished critical oversight, and opaque decision-making structures within geriatric care environments [30,43].

A central contribution of the framework is the operationalisation of governance intensity according to epistemic and functional risk. Rather than applying uniform oversight standards to all AI outputs, the SAIA-ETR architecture recognises that companionship prompts, medication reminders, behavioural recommendations, and evidence-based clinical claims entail substantially different ethical and clinical consequences.

The framework additionally contributes methodological innovation beyond conceptual synthesis alone. Specifically, it introduces measurable governance constructs, including the Societal Alignment Index and Trust Calibration Ratio, alongside escalation thresholds, structured audit pathways, and implementation-oriented governance mechanisms suitable for future empirical testing.

Furthermore, the framework does not advocate replacing professional judgement with automated systems. Instead, generative AI is conceptualised as epistemically constrained support infrastructure requiring context-sensitive verification, proportional oversight, and continuous human accountability.

This position is consistent with broader perspectives framing artificial intelligence as augmentative rather than substitutive clinical infrastructure, where human expertise remains central to safe and ethically legitimate healthcare delivery [17].

The proposed governance architecture may also contribute to ongoing debates concerning responsibility gaps in AI-mediated healthcare. Through the incorporation of RACI-based accountability structures, the framework clarifies governance responsibilities across developers, institutions, clinicians, carers, and auditors, thereby reducing ambiguity regarding oversight obligations and harm accountability.

Another important contribution concerns the explicit integration of age-related vulnerabilities into AI governance design. Current AI governance discussions frequently treat users as homogeneous populations, insufficiently accounting for the specific risks associated with frailty, dementia, sensory decline, low digital literacy, and social dependency. By incorporating vulnerability-sensitive governance escalation, the framework attempts to align AI deployment with the ethical imperatives of geriatric care and healthy ageing policy.

Nevertheless, several challenges remain. Governance-intensive frameworks may increase implementation burden, particularly in under-resourced healthcare systems lacking specialised AI governance infrastructures. Additionally, societal alignment itself may vary across cultural and institutional contexts, complicating universal operationalisation of value convergence metrics.

Pragmatic implementation will therefore likely require phased deployment strategies, institution-specific calibration, and governance proportionality mechanisms capable of adapting oversight intensity according to available clinical, technological, and human resources.

Future research should therefore prioritise longitudinal implementation studies, validation of alignment metrics, trust calibration experiments, cross-cultural governance comparisons, and empirical assessment of ageism-sensitive AI benchmarking protocols.

Importantly, the objective of the SAIA-ETR framework is not to eliminate uncertainty or automate ethical judgement, but to institutionalise proportional safeguards capable of reducing epistemic opacity, responsibility diffusion, and vulnerability amplification within healthy ageing ecosystems.

Ultimately, the governance of generative artificial intelligence in healthy ageing should not be understood solely as a technical challenge, but as a societal and epistemic question concerning whose values are represented, whose vulnerabilities are protected, and whose responsibilities remain enforceable within increasingly automated care environments.

### 5.1. Counter-Arguments and Risk Trade-Offs

Some may contend that tiered governance and SAIA evaluation could slow innovation or limit access to beneficial tools in under-resourced settings. However, unstructured deployment merely displaces burdens onto older adults, families, and frontline carers, often exacerbating inequities and digital exclusion [6]. The framework’s minimal pathway for resource-limited contexts preserves feasibility while upholding core protections.

### 5.2. Ethical Trade-Offs and Governance Tensions

The implementation of governance-intensive AI systems in healthy ageing environments may generate important ethical and operational tensions.

For example, increasing verification requirements and human oversight mechanisms may improve safety and accountability while simultaneously reducing scalability, accessibility, and responsiveness in resource-constrained care settings.

These governance requirements mirror international calls for independent auditing, lifecycle monitoring, and explicit human accountability in clinical AI deployment, particularly where system opacity intersects with high-stakes healthcare decision environments [11].

Similarly, highly protective governance structures designed to minimise harm may unintentionally reinforce paternalistic assumptions regarding older adults’ autonomy and decision-making capacity.

Tensions may also emerge between personalisation and privacy, particularly when AI systems require extensive behavioural, cognitive, or functional data to optimise adaptive interventions.

Accordingly, governance frameworks for healthy ageing should not be interpreted as mechanisms for eliminating ethical uncertainty, but rather as institutional structures intended to support proportionate, transparent, and context-sensitive decision-making under conditions of persistent epistemic limitation.

### 5.3. Limitations

Several limitations should be acknowledged.

First, the present framework remains primarily conceptual and therefore requires prospective empirical validation before large-scale institutional deployment. Although preliminary operational metrics and proof-of-concept pathways are proposed, their psychometric robustness and implementation reliability remain to be formally tested.

Second, the framework primarily addresses text-based large language models and may require adaptation for multimodal, autonomous, or continuously self-updating AI systems.

Third, societal alignment itself may vary across cultural, linguistic, socioeconomic, and healthcare contexts, potentially complicating universal standardisation of value convergence metrics.

Fourth, implementation feasibility may differ substantially across institutions depending on technological infrastructure, workforce capacity, regulatory maturity, and digital literacy levels among users and care professionals.

Finally, rapid advances in generative AI capabilities may alter the epistemic and governance assumptions underpinning the framework, necessitating continuous refinement and adaptive oversight mechanisms.

Despite these limitations, the SAIA-ETR framework provides a structured foundation for future empirical, ethical, and regulatory research concerning generative AI governance in healthy ageing ecosystems.

## 6. Conclusions

The present regulatory transition offers a narrow but decisive window for establishing governance standards capable of ensuring that generative AI strengthens, rather than undermines, dignity, autonomy, and functional ability in later life.

Without explicit governance mechanisms addressing societal alignment, epistemic legitimacy, calibrated trust, and accountability allocation, large language models risk reinforcing automation bias, benevolent ageism, epistemic opacity, and responsibility dilution in vulnerable older populations.

The SAIA-ETR framework was proposed as an integrated governance architecture designed to address these risks through multidimensional societal alignment assessment, epistemic classification, trust calibration mechanisms, and scalable responsibility allocation structures.

By operationalising governance intensity proportionally to clinical, ethical, and functional risk, the framework attempts to bridge the gap between abstract ethical principles and real-world implementation requirements in healthy ageing environments.

Crucially, the framework also advances the field by introducing preliminary measurable governance constructs, implementation pathways, escalation thresholds, and proof-of-concept validation strategies suitable for future empirical research.

Although substantial validation work remains necessary, the framework provides an initial operational foundation for integrating generative AI into healthy ageing contexts while preserving autonomy, dignity, equity, and functional ability among older adults.

Recent debates concerning healthcare artificial intelligence increasingly emphasise that ethical AI governance cannot rely exclusively on abstract principles or post hoc regulatory compliance mechanisms [14,22]. Instead, effective governance requires operationally actionable structures capable of integrating alignment evaluation, accountability allocation, trust calibration, and context-sensitive human oversight into real-world institutional practice.

Future empirical studies across culturally diverse and resource-variable geriatric settings will be essential to refine, validate, and operationalise the proposed governance architecture.

Only through deliberate, vulnerability-sensitive, and societally aligned governance can generative artificial intelligence genuinely contribute to healthy ageing without reproducing new forms of exclusion, dependency, or epistemic harm.

The governance of generative AI in healthy ageing will ultimately depend not only on technical capability, but on the capacity of institutions to align artificial intelligence systems with human dignity, epistemic responsibility, and vulnerability-sensitive care.

## Figures and Tables

**Table 1:** Operational Definitions Used in the SAIA-ETR Framework.

Concept	Operational Definition	Governance Relevance
Societal AI Alignment	Degree of convergence between AI-generated outputs and socially endorsed healthy ageing values, including autonomy, dignity, equity, and functional ability.	Supports evaluation of value consistency in geriatric AI systems.
Epistemic Authority	Perceived legitimacy and credibility attributed to AI-generated information within healthcare decision-making.	Influences reliance, trust calibration, and adoption behaviour.
Warranted Trust	Trust calibrated according to evidence of reliability, verifiability, contextual appropriateness, and reversibility.	Prevents inappropriate over-reliance on AI outputs.
Automation Bias	Tendency to over-rely on automated systems despite contradictory evidence or insufficient verification.	Particularly relevant in cognitively vulnerable older populations.
Digital Ageism	Technologically mediated discrimination, exclusion, or paternalistic assumptions directed at older adults.	May reinforce inequities and dependency within AI-assisted care.
Governance Intensity	Proportional degree of oversight, verification, auditing, and accountability mechanisms applied to AI outputs.	Enables risk-sensitive governance escalation.
Epistemic Placebo	Oversight mechanisms that create the appearance of safety without meaningful verification capacity.	Risks false reassurance and diluted accountability.
Functional Ability	Capacity to maintain activities and capabilities necessary for well-being in older age.	Central value target within healthy ageing governance.

**Table 2:** Preliminary interpretation thresholds.

SAI Score	Interpretation
4.0–5.0	High societal alignment
3.0–3.9	Moderate alignment requiring review
<3.0	Potentially misaligned output requiring escalation

**Table 3:** Preliminary thresholds.

TCR Value	Interpretation
>0.80	Appropriately calibrated trust
0.50–0.79	Moderate calibration requiring monitoring
<0.50	Potential over-reliance and governance concern

**Table 4:** Governance Escalation Across SAIA-ETR Tiers.

Tier	Output Type	Minimum Governance Requirement
Tier 1	Drafts, summaries, companionship prompts	Human review recommended
Tier 2	Reminders, checklists, adherence prompts	Source traceability mandatory
Tier 3	Suggestions, behavioural recommendations	Independent professional validation mandatory
Tier 4	Evidence claims, intervention recommendations	Formal institutional audit mandatory

**Table 5:** Comparative Characteristics of Major AI Governance Frameworks in Healthcare.

Framework	Primary Focus	Alignment Assessment	Epistemic Governance	Trust Calibration	Ageing-Specific Vulnerabilities	Responsibility Allocation
CONSORT-AI	Reporting quality	Limited	No	No	No	Limited
SPIRIT-AI	Clinical trial protocols	Limited	No	No	No	Limited
DECIDE-AI	Early clinical evaluation	Partial	Partial	Limited	No	Partial
WHO AI Guidance	Ethical principles	Partial	Partial	Limited	Limited	Partial
SAIA-ETR	Integrated governance architecture	Yes	Yes	Yes	Yes	Yes

Sources: [35,36,37].

## Data Availability

No new data were created or analysed in this study.
